# Breathing Exercises, Etc.

**Published:** 1888-07

**Authors:** John L. Davis

**Affiliations:** Professor of Therapeutics in the Medical College of the University of Southern California, Los Angeles, Cal.


### BREATHING EXERCISES IN THE PREVENTION AND TREATMENT OF LUNG DISEASES.

A paper read before the Los Angeles County Medical Society, April 6, 1888.

BY JOHN L. DAVIS, A. B., M. D.,

*Professor of Therapeutics in the Medical College of the University of Southern California, Los Angeles, Cal.*

There is no question that regular general exercise is of prime importance in maintaining the bodily organs and their functions in a state of health. This is one point upon which all physicians agree. Not only does exercise tend largely to the maintenance of health and general well-being, but it leads to that bodily vigor which resists disease. In other words, through properly regulated exercise, a reserve force is accumulated which may be drawn upon when needed.

This energy may be directed toward the development of special functions or organs, and one part or system of the body becomes conspicuously stronger or more active than the rest. It is continued exercise that produces the blacksmith's muscle, the touch of the blind, the dexterity of the juggler, the endurance of the athlete. Strong muscle, a sensitive touch, dexterity and endurance are all admirable elements, particularly in a sound body. But it is far too often the case, that the very foundation of strength and endurance and vigor is neglected ; and the lungs, which of all organs rank first in importance, whether we consider the functions of health or the danger of disease, are too apt to remain undeveloped; their fullest functional service is not carefnlly sought after.

There is no question that if the care that is given toward developing the muscular and nervous systems were devoted to strengthening the breathing apparatus and increasing lung capacity, an infinitely greater benefit would be obtained by the individual; a greater factor in preserving health and withstanding disease. This is especially the case with persons whose lungs are below par through weakness, either inherited or acquired.

In this connection there are three propositions, which hardly need demonstration:

1. In the ordinary individual the lungs are not fully developed; many of the air-cells have only to the slightest extent been brought into use. This fact is repeatedly illustrated in post mortem examinations of these organs.

2. Proper breathing and muscular exercises will bring these cells into use and enlarge the breathing capacity (*i. e.*, "*vital* capacity). By way of proof reference may be made to the effect of training in vocalists and athletes.

3. Individuals whose lungs are well developed are less liable to pulmonary diseases than are those whose lung capacity is less developed. In support of this proposition I may refer to the valuable paper of Dr. Balfour (Med. Chirurg. Trans., 1860, p. 263) in which he shows, from a large number of recruits for the English army, that among those whose lung capacity was below the average there was over four times the sickness that prevailed among recruits whose capacity was above average. One of the highest authorities upon the science of life insurance (Sieveking, Med. Adviser in Life Ins., p. 42) says: "Respiration and life may be regarded as synonymous, and we find that vital power may be measured by the manner in which the functions of respiration are carried on. Hence the stress that medical men, and even popular opinion, lays upon the value of a well developed chest, which affords an indication of the *vital capacity* of the lungs. In ordinary quiet respiration, the thorax is neither fully expanded nor fully emptied of the contained air. To measure its entire capacity—*i. e.*, to determine the whole amount of air which it is capable of taking in and discharging in one respiratory act—it is necessary that a forced inspiration and a forced expiration be made."

The average vital capacity is 225–250 cubic inches for a man of ordinary height at thirty years of age. The capacity increases with the individuals' height; and it also increases from the age of fifteen to thirty-five. In later life, however, it is found to decrease.

The average of expansion for the "normal" man is three inches; that is, the difference in chest circumference between the completest expiration and the fullest inspiration. If it falls much below this figure, life companies agree that the individual is an unsafe risk for insurance, because he is not likely to live out his "expectancy."

But systematic exercise will increase the expansion considerably. I have often examined patients and applicants for insurance whose expansion was over four inches, and in a few instances the expansion has reached five inches. In most, if not all cases of unusually large expansion, the individuals were either vocalists or players on wind-instruments, or they had taken special pains to develope their vital capacity. Some years ago, when I first made application for life insurance, my chest expansion was four inches; and this amount was (in a few weeks) increased to five inches by careful exercises, vocal and respiratory.

But the greatest benefits to be derived from lung exercise are not in the cases of healthy individuals, but rather in those whose vital capacity is below the normal—who are hollow-chested, stooping and feeble in their breathing The imperfect development of their respiratory function invites disease; their lungs are vulnerable. Proper exercise will throw off this debility and render them less liable to disease. We may go even a step further and say, that in many cases where lung disease actually exists, breathing exercise is one of the most valuable elements in treatment. I have often been gratified with the way in which a consolidated lung in chronic pneumonia of long standing and slow progress would improve under proper lung exercise. Indeed, in some of these cases it has seemed that properly regulated exercises have rendered greater service than could be derived from ordinary drugs.

The exercise which I have found of most value in developing the lungs may be described as follows:

Standing as erect as possible, with shoulders thrown back and chest forward, the arms hanging close to the body; the head up, with lips firmly closed, inhalation is to be taken as slowly as may be; at the same time the extended arms are to be gradually raised, the back of the hands upward, until they closely approach each other above the head. The movement should be so regulated that the arms will be extended directly over the head at the moment the lungs are completely filled. This position should be maintained from five to thirty seconds, before the reverse process is begun. As the arms are gradually lowered, the breath is exhaled slowly, so the lungs shall be as nearly freed from breath as possible at the time the arms again reach the first position, at the side. By these movements the greatest expansion possible is reached ; for, upon inspiration, the weight of the shoulders and pectoral muscles are lifted, allowing the thorax to expand fully; while upon exhalation, in lowering the arms, we utilize the additional force of this pressure upon the upper thorax to render expiration as complete as possible.

These deep respirations should be repeated five or six times ; and the exercise gone through with several times a day. It is hardly necessary to remark that the clothing must in no way interfere with the exercise.

In some cases this exercise is more advantageous when taken lying flat on the back, instead of standing. In this position the inspiratory muscles become rapidly strengthened by opposing the additional pressure exerted by the abdominal organs against the expanding lungs. And on the other hand, expiration is more perfect and full on account of the pressure of these organs. This is an exercise now advocated by several leading vocal teachers of Europe.

In conclusion, I will mention the exercises proposed by Dr. Dally (Bul. Gun. de Thésap., Sept. 20, 1881) for enlarging lung capacity :

"1. The first or normal is the vertical position, perfectly erect, as if standing against a wall, the arms hanging by the side. This position should be taken and kept ten minutes at a time, a number of times a day.

"2. The two arms and the hands are extended horizontally forward, the palms facing. The hands are separated slowly, whilst the chest is inclined forward. Remain in this position thirty seconds, and inspire deeply by the nose. Return to the initial position and expire. Execute this movement six times.

"3. The arms hang by the side ; raise them upward—the fingers well extended—above the head, the palms looking forward. Take a deep inspiration. Let fall the arms alongside the body, palms open, and expire slowly.

"4. Double rotation at the side. The subject being in the normal position (first), executes as large as possible, the arms well extended, double rotation laterally, and inclining the trunk forward each time that the arms are thrown behind, and never projecting the abdomen forward. This movement is executed entirely by the scapulo-humeral articulation.

"5. The arms are crossed horizontally, the palms looking backward. Flexion lateral, alternately of the trunk. The flexion will be then regular, transverse, the abdomen drawn in, the legs extended apart, the pelvis fixed. The limit of the flexion is the vertical position of the elevated arm. Mild inspiration during the flexion, at its terminal expiration. Execute these movements six or eight times.

"These exercises, if faithfully carried out, improve the shape and capacity of the thorax and check the development of incipient phthisis.

"According to Dr. Dally, dyspnœa, polysarca, and arthritic conditions are removed or sensibly ameliorated. Venous stases, varicose dilatations, and infarctions are, after some weeks of such movements, much improved, when the circumstances are favorable. The great obstacles to this hygienic medication in our civilization are the habitual laziness and idleness, and the indisposition to devote time and interest to such means."

